# Rhodiola rosea: A Therapeutic Candidate on Cardiovascular Diseases

**DOI:** 10.1155/2022/1348795

**Published:** 2022-02-27

**Authors:** Yingqing Chen, Minli Tang, Shuo Yuan, Shuang Fu, Yifei Li, You Li, Qi Wang, Yuying Cao, Liping Liu, Qinggao Zhang

**Affiliations:** ^1^Chronic Diseases Research Center, Dalian University College of Medicine, Dalian, Liaoning 116622, China; ^2^Yanbian University, Yanji, Jilin 133022, China; ^3^Zhongshan Hospital of Dalian University, Dalian, Liaoning 116001, China

## Abstract

Cardiovascular diseases, also known as circulatory diseases, are diseases of the heart and blood vessels, and its etiology is hyperlipidemia, thick blood, atherosclerosis, and hypertension. Due to its high prevalence, disability, and mortality, it seriously threatens human health. According to reports, the incidence of cardiovascular disease is still on the rise. Rhodiola rosea is a kind of traditional Chinese medicine, which has the effects of antimyocardial ischemia-reperfusion injury, lowering blood fat, antithrombosis, and antiarrhythmia. Rhodiola rosea has various chemical components, and different chemical elements have the same pharmacological effects and medicinal values for various cardiovascular diseases. This article reviews the research on the pharmacological effects of Rhodiola rosea on cardiovascular diseases and provides references for the clinical treatment of cardiovascular diseases.

## 1. Introduction

Cardiovascular disease (CVD) is the general term for heart and vascular diseases, mainly including ischemic heart disease, cerebrovascular disease, peripheral vascular disease, and rheumatic heart disease. Cardiovascular disease is a global epidemic with 80% of the disease burden in low-income and middle-income countries [1]. The mortality of cardiovascular disease is increasing. In China, the annual number of deaths owing to CVD increased from 2.51 million to 3.97 million between 1990 and 2016; the prevalence rate of CVD overall increased significantly from 1990 to 2016 by 14.7% [2]. In Europe, CVD causes 49% of mortality, which is the most important cause of premature mortality and disability adjusted life years (“DALYS”) in Europe. Approximately € 192 billion is the cost of annual health care for CVD in the European Union [3]. CVD has various complex causes and forms of the disease, and it is difficult to treat, which accounts for the first cause of death in the world, about 41%. Hypertension, diabetes, dyslipidemia, obesity, overweight, smoking, and physical inactivity are important influencing factors of CVD [3, 4]. Rhodiola rosea is a perennial herb of the Rhodiola family. According to the Global Biodiversity Information Facility, 136 Rhodiola species are recorded. In China, there are 73 Rhodiola species [5]. As a worldwide plant adaptogen [6], Rhodiola rosea can improve cardiovascular and cerebrovascular system functions. The structure of different chemical components of Rhodiola rosea is different, and its pharmacological effects and medicinal value for various cardiovascular diseases are also other. This article reviews the pharmacological effects of Rhodiola rosea and its active ingredients on cardiovascular diseases in the past ten years.

## 2. The Main Ingredients of Rhodiola rosea

The main active ingredients of Rhodiola rosea include salidroside (Sal), gallic acid, tyrosol, rosavin, rosaline, etc. The specific composition of the components is shown in Table 1.

## 3. Protective Effects of Rhodiola rosea on Myocardial Ischemia-Reperfusion Injury (MIRI)

### 3.1. Protective Effects on Vascular Endothelial Cells

Vascular endothelial injury begins in the early stage of reperfusion and gradually aggravates. In the early phase of reperfusion, it activates vascular endothelial cells, increases the secretion of adhesion molecules, promotes the adhesion of leukocytes to them, and produces reactive oxygen species (ROS), causing lipid peroxidation, which prevents endothelial cells from secreting vascular relaxing factors and increases the secretion of vasoconstrictive substances, thereby constricting the coronary arteries and coagulating platelets, obstructing coronary blood flow and aggravating myocardial damage.

**Figure 1:**
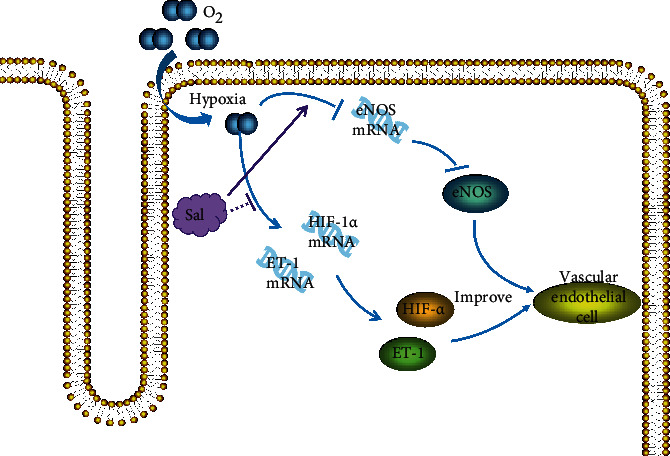
Mechanism of salidroside improving the diastolic and systolic function of vascular endothelium in myocardial ischemia-reperfusion injury model. Salidroside promotes the expression of eNOS by inhibiting HIF-1*α* and ET-1 produced during tissue hypoxia and improving the vasoconstriction function of vascular endothelium.

Studies have shown that salidroside can effectively improve the vasoconstrictor function of vascular endothelium and promote the angiogenesis of ischemic myocardium in rats by affecting the expression of vascular endothelial growth factor (VEGF), promote the angiogenesis of ischemic myocardium in rats, and protect the myocardial ischemia-reperfusion injury. Gao and others used salidroside to intervene in the endothelial cell line of a hypoxia model and tested vascular endothelial cell hypoxia-inducible factor 1*α* (HIF-1*α*), endothelin-1 (ET-1), and vascular endothelial cell NO synthase (eNOS) gene expression. It is concluded that salidroside inhibits the gene expression of HIF-1*α* and ET-1 and promotes the expression of eNOS, as well as the expression of vascular endothelial contractile factors. Salidroside promotes the expression of vascular endothelial relaxing factors and improves vascular endothelial function [14]. Gao et al. [15] investigate the effects of Rhodiola rosea on angiogenesis and expressions of hypoxia-inducible factor 1alpha (HIF-1*α*), hypoxia-inducible factor 1beta (HIF-1*β*), and vascular endothelial growth factor (VEGF) in the myocardium of rats with acute myocardial infarction (AMI). They found that Rhodiola rosea may promote angiogenesis in the myocardium of rats with AMI through elevating the expressions of HIF-1*α*, HIF-1*β*, and VEGF. Rhodiola rosea increases the expressions of HIF-1*α*, HIF-1*β*, and VEGF during ischemia or hypoxia. Leung et al. [16] study the effect of salidroside on aortic function in rats and found that salidroside is effective in preserving the NO bioavailability and thus protects against homocysteine-induced impairment of endothelium-dependent relaxations, mainly through inhibiting the NOX2 expression and ROS production. The relevant mechanism of action is shown in Figure 1.

### 3.2. Antioxidative Stress Properties

When myocardial ischemia-reperfusion occurs, the myocardium will be ischemic and hypoxic. Oxygen-free radicals (OFR) will be produced in the ischemic part. The body's antioxidant activity will decrease, and the antioxidant capacity will be insufficient, causing oxidative stress. The cardiomyocytes are acidic, and Ca^2+^ enters the cells to balance the pH, causing intracellular calcium overload and promoting the strengthening of the inflammatory response of white blood cells and the lack of high-energy phosphate compounds, which in turn leads to cell damage and even death.

**Figure 2:**
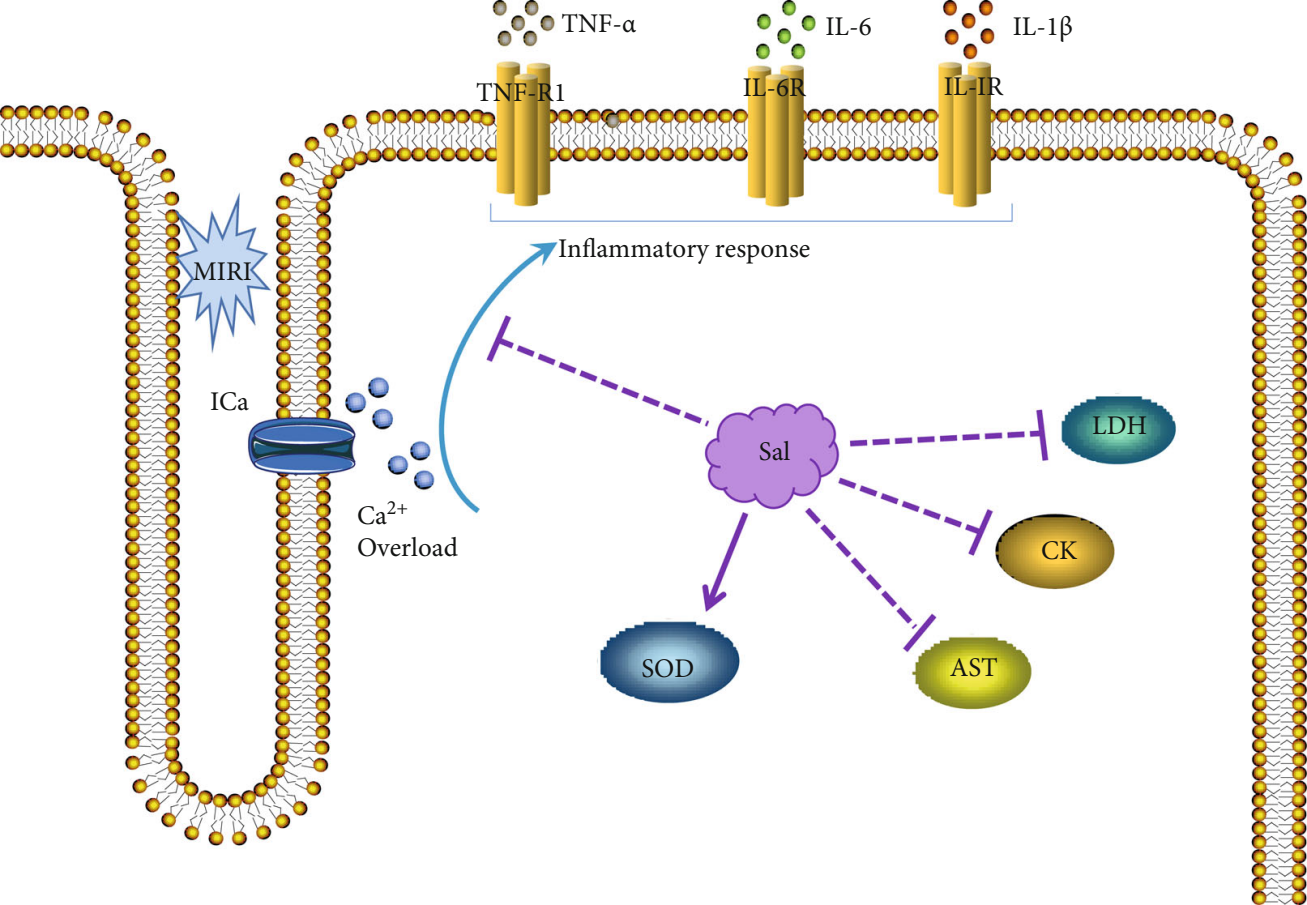
Mechanism of salidroside on antioxidative stress after myocardial ischemia and reperfusion. Salidroside can inhibit LDH, CK, AST, and the infiltration of inflammatory cells, promoting the expression of SOD against oxidative stress.

Gupta et al. [17] showed that Rhodiola rosea extracts not only inhibited malondialdehyde (MDA) and lactate dehydrogenase (LDH) in the blood, liver, and muscle of rats but also increased the activities of reducing glutathione (GSH) and superoxide dismutase (SOD) in the blood. Thus, the oxidative capacity of rats during cold (5 degrees C)-hypoxia (428 mmHg)-restraint (C-H-R) exposure (C-H-R) exposure and poststress recovery can be improved, and the membrane permeability can be maintained. As shown in Figure 2, salidroside can significantly reduce the activity of CK and LDH in the serum of rats after MIRI and reduce the levels of tumor necrosis factor-*α* (TNF-*α*), interleukin-1*β* (IL-*β*), and interleukin-6 (IL-6) in cells [18]. In terms of pathology, the administration of salidroside can attenuate the overall degree of myocardial pathology, the infiltration of inflammatory cells, and the local focal necrosis.

### 3.3. Interference of Cardiomyocyte Apoptosis

Countless studies have shown the association and or causation between the apoptotic loss of myocytes and the progression of heart diseases, including heart failure, in human and animal models [19, 20]. Generally, there are three ways to affect cell apoptosis: mitochondrial pathway, endoplasmic reticulum signaling pathway, and death receptor pathway.

**Figure 3:**
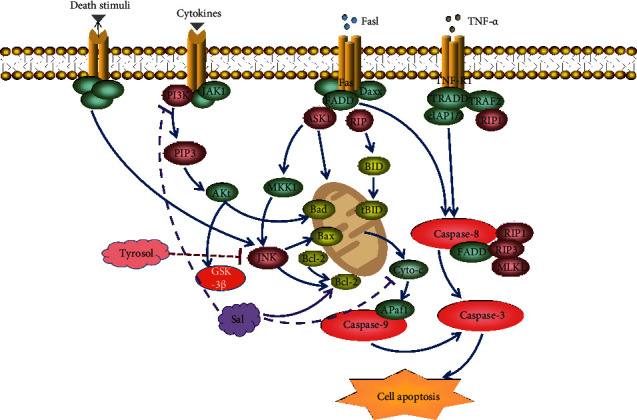
Mechanism of salidroside and tyrosol on cardiomyocyte apoptosis pathway. Salidroside can inhibit the release of Cyto-C from mitochondria to the cytoplasm by promoting the expression of Bcl-2 and Bax and subsequently inhibiting a series of downstream caspase-3 and caspase-9 protein activities from inhibiting cardiomyocyte apoptosis. Moreover, salidroside can activate the PI3K/Akt pathway followed by the upregulation of p-Akt and p-GSK-3*β* and protects myocardial cells to achieve protection against myocardial ischemia-reperfusion injury.

Zhong et al. [21] confirmed the protective effect of salidroside on mitochondrial damage from the four aspects, including cardiomyocyte apoptosis rate, mitochondrial membrane potential, degree of mitochondrial division, and cytochrome C (Cyto-C) expression in mitochondria. The expression level of Cyto-C in mitochondria is related to the opening degree of mitochondrial membrane permeable pores. Proteins related to apoptosis include the B-cell lymphoma-2 gene (Bcl-2) family, namely, the antiapoptotic protein Bcl-2 and the proapoptotic protein Bcl-2-associated X (Bax). Besides that, it also includes the death receptor-mediated apoptosis pathway initiator Fas, the key initiation factor caspase-8, caspase-3, caspase-9, mitochondrial-mediated apoptosis pathway initiator Cyto-C, and so on. Multiple studies have shown that salidroside can inhibit the release of Cyto-C from mitochondria to the cytoplasm by promoting the expression of Bcl-2 protein, inhibiting the expression of Bax protein, and subsequently inhibiting a series of downstream caspase-3 and caspase-9 protein activities and realize the inhibitory effect on cardiomyocyte apoptosis [22–24]. The PI3K-Akt signal transduction pathway is important to protect cardiomyocytes. When PI3K is activated, it will form phosphorylated inositol phospholipid product PIP3 and then activate downstream substances to cause the expression of signal factors such as the Bcl-2 family and glycogen synthase kinase (GSK-3*β*) [25–28]. Several studies have shown that the phosphorylation of GSK-3*β* and Akt in cells, after salidroside treatment, is significantly enhanced and which is significantly reduced after the administration of PI3K inhibitors, so it can be proved that salidroside can activate the PI3K/Akt pathway followed by the upregulation of p-Akt and p-GSK-3*β*, and protects myocardial cells to achieve protection against myocardial ischemia-reperfusion injury [29]. In addition, Sun et al. [30] used the MIRI-treated H9C2 rat cardiomyocyte model to check the influence of tyrosol on the levels of ROS, heat shock protein 70 (Hsp70), extracellular signal-regulated kinase (EPK), c-Jun amino-terminal kinase (JNK), Bcl-2, Bax, and caspase-8. It is known that the cardioprotective effect of tyrosol is related to the reduction of ROS; the inhibition of EPK, JNK, and caspase-8 activation; the increase of Hsp70; and the ratio of Bcl-2/Bax. The relative mechanism is shown in Figure 3.

### 3.4. Protective Effects on Mitochondrial Damage

**Figure 4:**
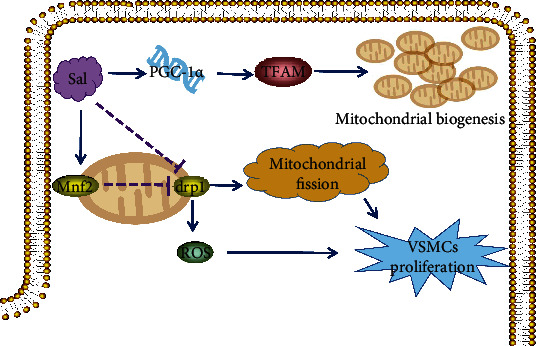
Mechanisms of salidroside on inhibiting VSCM proliferation via promoting mitochondria biogenesis and interfering with mitochondrial fission. Salidroside can promote PGC-1*α*, TFAM, and Mnf2 and inhibit Drp1 to inhibit VSCM proliferation.

Mitochondria is essential for maintaining cardiac homeostasis by providing the major energy required for cardiac excitation-contraction coupling and controlling the intracellular and death pathways [31]. Mitochondrial damage extension suggested as a critical factor for determining myocardial injury under MIRI toward progression to heart failure [32, 33]. MIRI could significantly increase mitochondrial permeability and which resulted in dissipation of electron and proton gradients, dysregulation of mitochondrial calcium homeostasis, and release of superoxide to cause myocardial cell death [34, 35]. One previous study suggested that salidroside could attenuate MIRI-induced myocardial apoptosis via preserving mitochondrial transmembrane potential and enhancing Bcl-2/Bax ratio [21]. Furthermore, Zhuang and his colleagues checked the effect of salidroside on vascular smooth muscle cell (VSMC) proliferation, ROS generation, and mitochondrial dynamics under the challenge of high glucose. They found that salidroside could inhibit VSMC proliferation, dynamin-related protein 1 (Drp1) expression, and mitochondrial ROS level, while upregulate Mitofusin 2 (Mfn2) expression [36]. These results suggested that salidroside might play its therapeutic effect on diabetic cardiomyopathy via maintaining mitochondrial dynamic homeostasis and regulating oxidative stress. In addition, the protective effect of salidroside on diabetic cardiomyopathy is partially associated with the activation of mitochondrial SIRT3, AMPK/Akt, and PGC-1*α*/TFAM and subsequent improving mitochondrial biogenesis and its function [37]. These findings suggest that salidroside exhibits potential to be a promising drug for preventing and treating ischemic and diabetic myocardial diseases through the prevention of mitochondrial damage and its function (Figure 4).

## 4. Resisting Myocardial Fibrosis and Heart Failure (HF)

**Figure 5:**
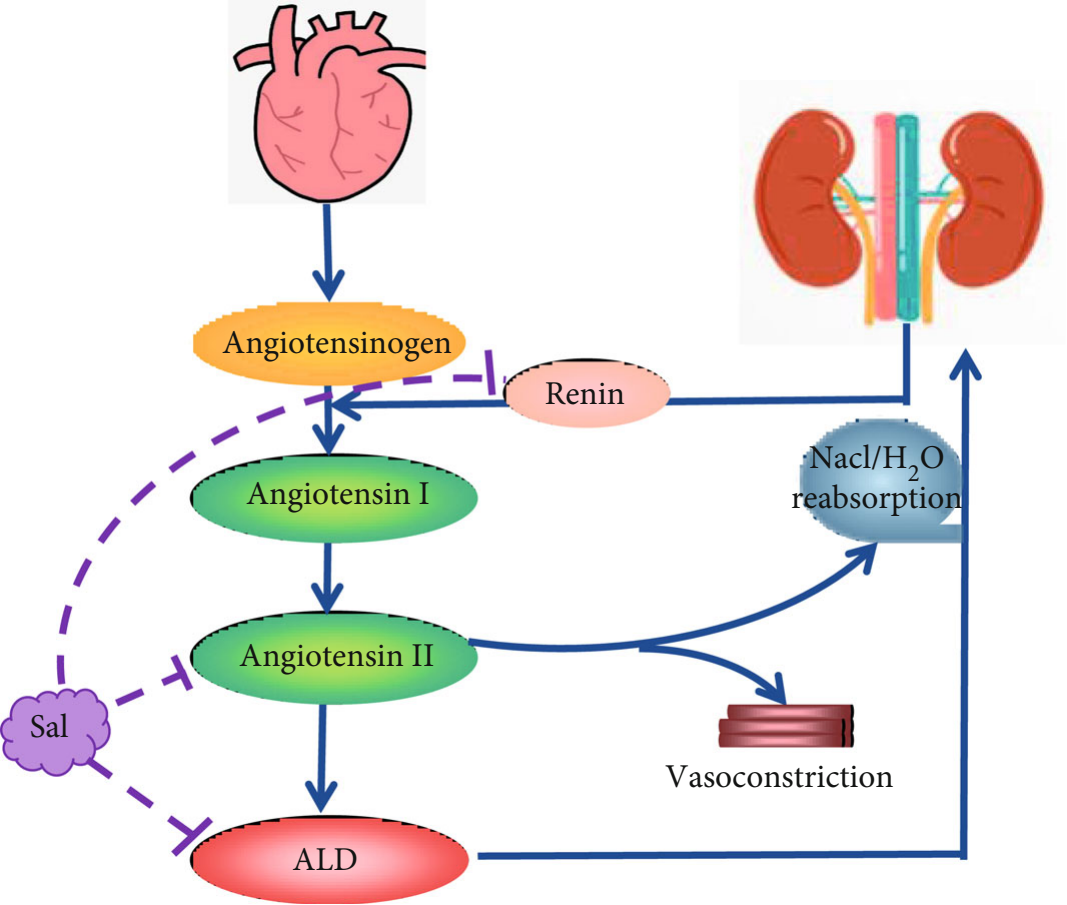
Salidroside participates in the mechanism of resisting heart failure by inhibiting the RAAS system.

Chen et al. [38] found that Sal might protect against myocardial fibrosis in mice with myocardial infarction (MI) by decreasing the expressions of TNF-*α*, TGF-*β*1, IL-1*β*, and Bax and increasing Bcl-2, VEGF, Akt, and eNOS. In addition, some research also found that Sal significantly upregulated the expression of HO-1 and improved the cardiac dysfunction myocardial hypertrophy and myocardial fibrosis induced by diabetes in mice [39]. The Renin-angiotensin-aldosterone system (RAAS) is involved in the occurrence and development of heart failure and is one of the main factors affecting ventricular remodeling. As shown in Figure 5, Wu et al. [40] found that salidroside might prevent cardiac function and ventricular remodeling in rats with heart failure by inhibiting the activation of the RAAS system in HF. In addition, the ethanol extract of Rhodiola rosea was also reported to increase the expression of peroxisome proliferator-activated receptor (PPAR), to enhance the cardiac output of diabetic heart failure rats, and to increase the myocardial contractility to combat heart failure [41]. Hsiao et al. [42] found that Rhodiola rosea can inhibit the expression of IL-17 and its downstream target genes, thereby reducing the levels of fibrosis and apoptosis and inhibiting ventricular arrhythmia.

### 4.1. Hypolipidemic Function

**Figure 6:**
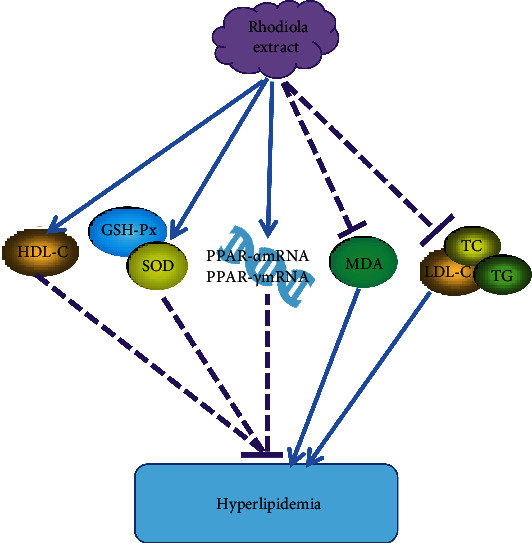
Hypolipidemic mechanism of salidroside. The ethanol extract of Rhodiola rosea angustifolia can reduce blood fat by affecting the absorption, transportation, antioxidation of cholesterol, and the activation of PPAR-*α* and PPAR-*γ* receptors.

The clinical manifestation of hyperlipidemia is high blood lipid levels, often accompanied by various of complications. The leading diagnostic indicators are total cholesterol (TC), triglycerides (TG), low-density lipoprotein cholesterol (LDL-C), and high-density lipoprotein cholesterol (HDL-C) [43]. As shown in Figure 6, Shin et al. [44] used the ethanol extract of Rhodiola rosea angustifolia to gavage a high-fat mouse model, and the results showed that it can significantly reduce the content of TC, TG, and LDL-C and increase the content of HDL-C in the hyperlipidemia model mice. Simultaneously, it can increase the activity of GSH-Px and SOD and reduce the content of malondialdehyde (MDA). It shows that the ethanol extract of Rhodiola rosea angustifolia can reduce blood fat by affecting the absorption, transportation, and the antioxidation of cholesterol. Wang [45] found that Rhodiola rosea extract can effectively reduce the body weight (BW) of rats and improve blood sugar (FBG), blood pressure (BP), and dyslipidemia in rats. The expression of liver peroxisome proliferator-activated receptors (PPAR-*α* mRNA, PPAR-*γ* mRNA) in the Rhodiola group was significantly increased because PPAR-*γ* is closely related to fatty differentiated cells, lipid metabolism, glucose metabolism, and inflammation. Therefore, the effect of Rhodiola rosea extracts on protection against glucose and lipid metabolism disorders may be related to the activation of PPAR-*α* and PPAR-*γ* receptors.

### 4.2. The Protective Effect on Thrombosis

There are mainly three well-known causative factors of thrombosis: (1) Vascular endothelial injury triggers coagulation factors and collagen in the connective tissue and blood to promote coagulation, causing platelets to adhere to the thrombus. (2) The blood flow is slow and stagnated due to the change of the vascular state, and the endothelial cells die due to hypoxia. (3) The hypercoagulable state of blood easily forms a thrombus.

Zhang et al. [46] used the in vitro thrombosis method and established a blood stasis rat model to observe the effects of salidroside on coagulation-related indicators such as the length of the in vitro thrombus, dry and wet weight, and blood viscosity. After the treatment of salidroside, thrombus length was significantly shortened; dry weight and wet weight were reduced considerably; hematocrit, high shear viscosity, and the low shear viscosity of the blood were inhibited considerably; and platelet aggregation rate was significantly reduced. It shows that salidroside has a significant inhibitory effect on the formation of in vitro thrombosis, improves blood rheology, and can prolong the clotting time. Liu and Jiang [47] used the Dazhu Rhodiola Rosea Capsule to treat patients with unstable angina pectoris and reached the same conclusion. However, there was no significant difference in the results in mouse body weight, four items of coagulation, red blood cells, white blood cells, platelet count (PLT), and in mean platelet volume (MPV) detected by Liu (*P* > 0.05). It is suggested that the effect of salidroside and antithrombosis may not be directly related to the coagulation system. He also counted the bleeding time of tail docking and proved that salidroside could prolong bleeding time. Carrageenan is a kind of polysaccharide with a strong inflammatory effect. Subcutaneous or intraperitoneal injection of carrageenan can cause thrombus formation in the tail of mice, which is more intuitive for evaluating antithrombotic drugs. Liu used the intraperitoneal injection of carrageenan to construct thrombosis mouse model and measured the black tail rate and length. He injected salidroside (20 mg/kg) into the mouse intraperitoneally and then measured tail bleeding time, arterial thrombus, and venous thrombosis. These results showed that salidroside has the effect of preventing thrombosis.

**Figure 7:**
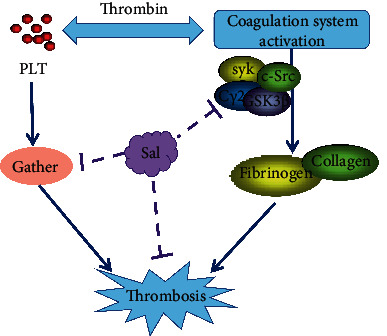
Antithrombotic mechanism of salidroside on AKT/GSK3*β* signaling pathway. Salidroside can inhibit the expression of syk, c-Src, PLC*γ*2, GSK3*β*, and platelet aggregation from inhibiting thrombosis.

As shown in Figure 7, Wei et al. [48] studied human and mouse platelets by treating with salidroside. Salidroside inhibits thrombin or CRP-induced platelet aggregation and adenosine triphosphate release but does not affect the expression of P-selectin and glycoproteins (GP). The platelets treated with salidroside have reduced spreading on fibrinogen and collagen and reduced clot retraction. The phosphorylation of proto-oncogene (c-Src), skin tyrosine kinase (Syk), and phospholipase C*γ*2 (PLC*γ*2) was reduced. In thrombin-stimulated platelets, salidroside inhibits the phosphorylation of AKT (T308/S473) and glycogen synthase kinase 3*β* (GSK3*β*). The addition of the GSK3*β* inhibitor reversed the inhibitory effect of salidroside on platelet aggregation and clot retraction. In short, salidroside inhibits platelet function and thrombosis through the interference of the AKT/GSK3*β* signaling pathway. It suggests that salidroside may be a new type of therapeutic drug for treating thrombosis and cardiovascular diseases.

### 4.3. Antiarrhythmic Effect

**Figure 8:**
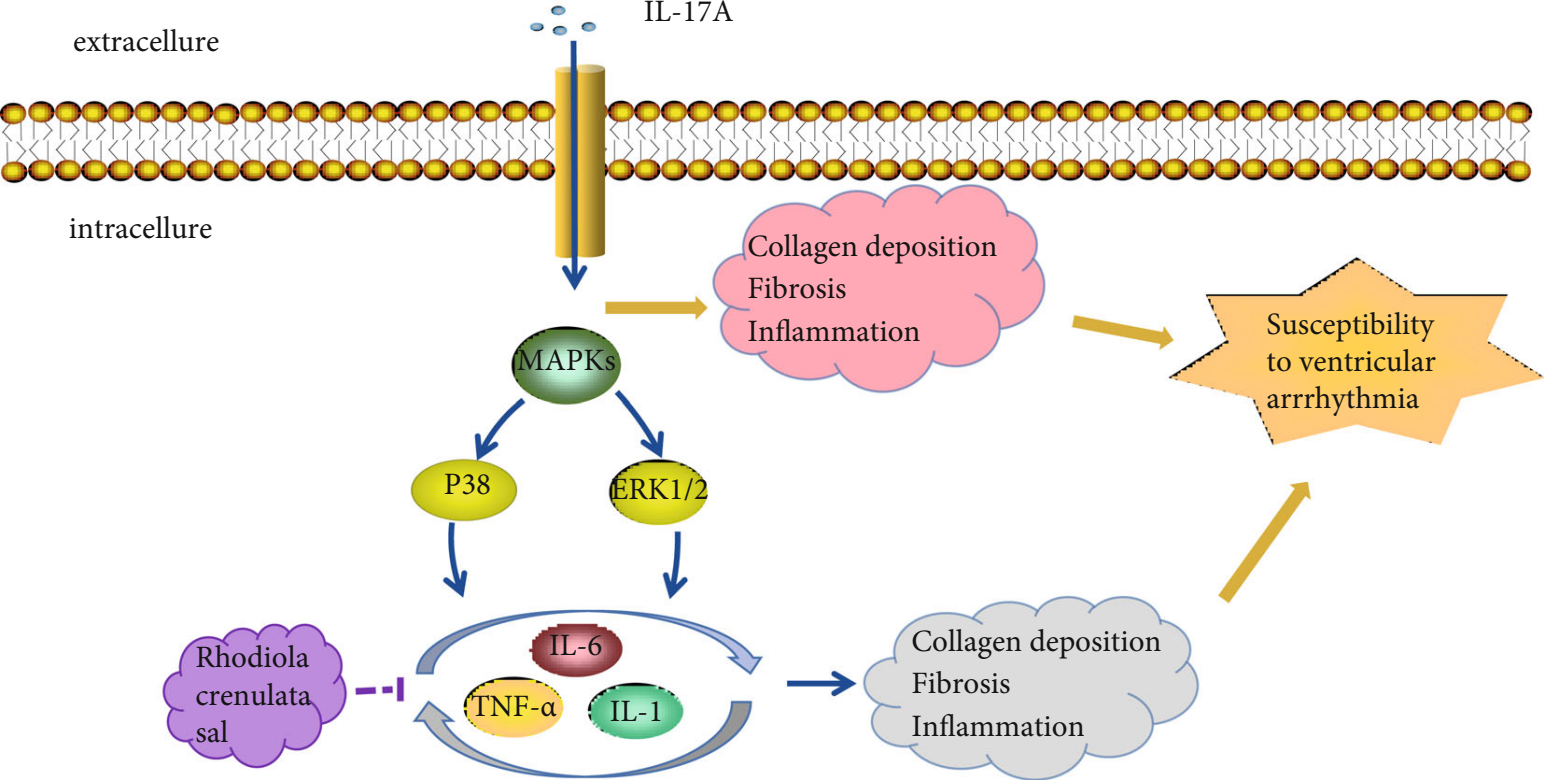
The mechanism for the protective effects of RC in ventricular arrhythmia. RC inhibits cardiac fibrosis by inhibiting the expression of IL-6, TNF-*α*, IL-1*β*, and IL-8 from achieving the purpose of interfering arrhythmia.

Arrhythmia is a common cardiovascular disease, which is very harmful and may lead to sudden death. By studying the molecular mechanism and electrophysiological properties of salidroside in inhibiting atrial fibrillation, Liu et al. [49] detected the monophasic action potential, histology, ion channels, and PI3K/AKT/eNOS. Rhodiola rosea can stabilize the ion pumps and calcium channels on the cell membrane, rebuild the atrial potential of rabbits with heart failure, reduce atrial fibrosis, inhibit atrial fibrillation, and eliminate ectopic rhythm. The beneficial electrogenic effects of Rhodiola rosea may be related to voltage-gated potassium channel proteins (Kv1.4, Kv1.5, Kv4.3, KvLQT1) and calcium channel proteins (Cav1.2), and myocardial sarcoplasmic reticulum Ca^2+^-ATP Enzyme 2a (SERCA2a) is involved in the activation of PI3K/AKT channels. As shown in Figure 8, Hsiao et al. [42] used Rhodiola rosea, and Sal treatment in HF left ventricle in rabbit and found that (1) the levels of WBC and CD4 T cells were decreased; (2) the expression of IL-17 and its downstream target genes, IL-6, TNF-*α*, IL-1*β*, IL-8, and CCL20, was reduced; (3) the level of NLRP3 inflammasome was decreased; (4) fibrosis and collagen production were significantly downregulated; (5) p38 MAPK and ERK1/2 phosphorylation were attenuated; (6) the inducibility of VA was decreased; and (7) the levels of Kir2.1, Nav1.5, NCX, PLB, SERCA2a, and RyR were upregulated. RC inhibited the expression of IL-17 and its downstream target genes mediated by activation of several MAPKs, which decreased the levels of fibrosis and apoptosis and suppressed VA. Rhodiola rosea reduces ventricular arrhythmias by inhibiting IL-17 activation and MAPK signaling in the rabbit.

### 4.4. Adjuvant Treatment of Hypertension

Persistent abnormal blood vessel function can lead to worsening hypertension and target organ damage. In spontaneously hypertensive male rats, Lee et al. [50] recorded the systolic blood pressure (SBP) and heart rate of male spontaneously hypertensive rats by the tail-cuff method and measured the plasma *β*-endorphin content by enzyme-linked immunosorbent assay. They found that the water extract of Rhodiola rosea could induce the release of *β*-endorphin and reduce the systolic blood pressure of spontaneously hypertensive rats. Rhodiola rosea water extract can induce *β*-endorphins in spontaneously hypertensive rats to reduce systolic blood pressure (SBP). Long-term salidroside intervention therapy can attenuate blood pressure by dilating resistance blood vessels and volume blood vessels and vascular dysfunction, as well as inhibiting vascular remodeling [51, 52].

Essential hypertension is not only caused by hemodynamic disorders and is also associated with blood lipid metabolism. Yang et al. [53] elaborated on the mechanism of Rhodiola rosea on left ventricular remodeling in hypertensive rats; that is, AngII played an essential role in left ventricular remodeling and participated in the synthesis and release of aldosterone (ALD). ALD can interact with fibroblast cells. The mineralocorticoid receptor in the plasma regulates the synthesis of type I and type III collagen. Rhodiola rosea can inhibit the synthesis of AngII and ALD, thereby promoting left ventricular remodeling.

### 4.5. Protective Effect on Hyperglycemia

**Figure 9:**
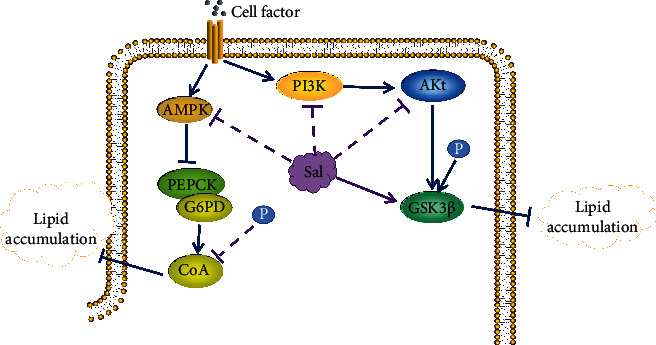
Mechanism of salidroside improving blood glucose. Rosea inhibits PEPCK and G6PD by activating AMPK, leading to reduced phosphorylation of CoA to reduce lipid accumulation, and Sal also reduces lipid accumulation in peripheral tissues by increasing phosphorylation of GSK3*β*.

High blood sugar can easily lead to coronary atherosclerosis, leading to cardiovascular diseases such as coronary heart disease. Therefore, improving blood sugar can effectively inhibit cardiovascular illness. As shown in Figure 9, research has found that salidroside can regulate the level of blood glucose and insulin in patients with diabetes and cerebral infarction [54–60]. Salidroside induces the phosphorylation of adenylate-activated kinase (AMPK) and PI3K/AKT and increases GSK3*β* in hepatocytes in a dose-dependent manner. AMPK activation inhibits the expression of phosphoenolpyruvate carboxy kinase (PEPCK) and glucose-6-phosphatase (G6PD), leading to the phosphorylation of acetyl CoA, reducing lipid accumulation in peripheral tissues, and affecting insulin metabolism in multiple ways. In isolated mitochondria, salidroside can inhibit respiratory chain complex 1, disrupt the oxidation/phosphorylation coupling, and depolarize the mitochondrial membrane potential. Eventually, the body's internal AMP/ATP ratio temporarily increases, thereby significantly reducing blood sugar and serum insulin levels. At this level, it can alleviate insulin resistance and play an antidiabetic effect [59]. The polysaccharides in Rhodiola rosea extract have a two-way regulating effect on blood sugar. It can inhibit the increase of liver glycogen caused by glucose, enhance the decomposition of liver glycogen caused by alloxan and epinephrine, and reduce the hyperglycemia caused by epinephrine. It can also improve hypoglycemia caused by insulin [61]. The hypoglycemic effect of Rhodiola rosea polysaccharide may be due to the reduction of pancreatic tissue damage, improvement of pancreatic tissue morphology, and increased insulin secretion [62, 63]. Besides, many documents show that Rhodiola rosea polysaccharides cannot lower blood sugar after being absorbed through the gastrointestinal tract. However, intramuscular, intraperitoneal, and intravenous injection of Rhodiola rosea polysaccharide can produce significant hypoglycemic effects.

### 4.6. Antiaging Effect of Rhodiola rosea on Cardiac Diseases

**Figure 10:**
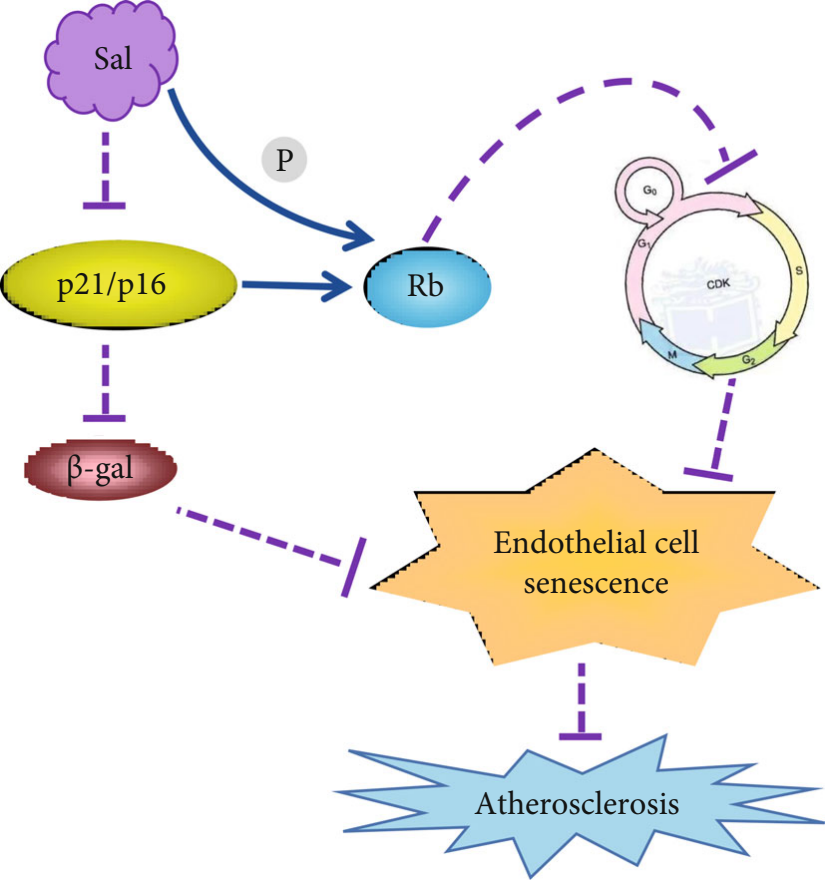
Salidroside protects against atherosclerosis (AS) by attenuating cellular senescence. Salidroside can inhibit the expression of P21 and P16 to promote the cell cycle from G0/G1 phase to S phase and upregulated *β*-gal, so that inhibits AS.

Aging is recognized as an independent risk factor for the development of cardiovascular diseases such as atherosclerosis and heart failure. The fundamental mechanism of aging is the accumulation of senescent cells and which appears to play a crucial role in the process of cardiovascular disease through the secretion of senescence-associated secretory phenotype (SASP) [64]. SASP is a trigger of chronic inflammation, oxidative stress, and decreased nitric oxide resulting in age-associated cardiovascular damage [64, 65]. Recently, growing evidence showed that the epigenetics also exhibited an important role in the chronic inflammation, cellular senescence, and oncogenesis [66–68]. Several studies showed the beneficial effects of Rhodiola rosea on lifespan extension in numerous model organisms, such as fruit flies [69], worms [70], and yeast [71] without interfering with daily food intake and fecundity. One previous study clearly demonstrated that salidroside could slow down the process of human umbilical vein cell senescence by regulating the cell cycle in an atherosclerosis model to clarify the relationship between endothelial cell senescence and atherosclerosis [72]. Sun and his colleagues declared that the possible mechanism is that salidroside might promote the phosphorylation of retinoblastoma protein (Rb) by downregulating the expression of p16, p21, and p66, thus triggering the cell cycle from G0/G1 phase to S phase [72]. In addition, more recent study showed that salidroside could prevent endothelial cell senescence by establishing hyperhomocysteine (HHcy) mouse model through downregulating CD68 and intercellular adhesion molecule 1 (ICAM1) to reduce the activity of senescence-related protein- (SA-) *β*-gal. Conversely, salidroside can enhance Sirt3 in aorta-derived endothelial cells to prevent them from premature senescence [73]. Overall, these findings strongly demonstrate that the active component of Rhodiola rosea can protect against cardiac diseases through the amelioration of cellular senescence and which provides a novel therapy for the treatment of diverse cardiomyopathy (Figure 10).

### 4.7. Anti-inflammatory Effect of Salidroside on Cardiac Diseases

**Figure 11:**
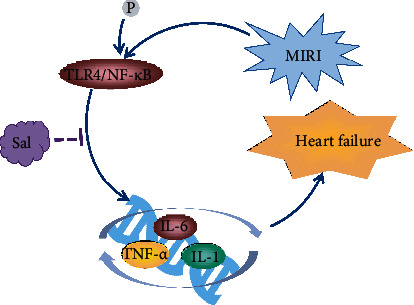
The anti-inflammatory effect of salidroside on preventing cardiovascular disease. Salidroside downregulating the expression of IL-6, IL-1, and TNF-*α* by inhibiting NF-*κ*B signaling pathway, thus protecting against myocardial infarction and heart failure.

Inflammation plays an important role in both chronic disease and cardiovascular disease, and low-grade inflammation can result in intracellular and mitochondrial oxidative stress that is associated with most cardiovascular disease, representing a cardiovascular risk factor that can be targeted by pharmacological treatment [74, 75]. In the MIRI model, salidroside could attenuated the proinflammatory cytokines including tumor necrosis factor-*α* (TNF-*α*), interleukin- (IL-) 1*β*, and IL-6 in serum by inhibiting TLR4/NF-*κ*B signaling pathway, and that could ameliorate cell apoptosis and the heart failure assessed by histopathological examination and TUNEL assay [24]. In addition, Zhu and his colleagues also investigated the protective effect of salidroside on isoproterenol- (ISO-) induced myocardial ischemia. They found that salidroside could increase SOD activity and decrease NOX4, NF-*κ*B p65, and AP-1 expression in the heart. These results revealed that salidroside might be a potential treatment for ischemic heart disease through attenuating the inflammatory response [18]. Taken together, inflammation is undoubtedly relative to the development of cardiovascular diseases, while salidroside possessed a strong anti-inflammatory effect and which might be a potential for treating chronic cardiac diseases such as MIRI and heart failure (Figure 11).

## 5. Conclusion

As a worldwide plant adaptogen, Rhodiola rosea is widespread throughout the world. Rhodiola rosea has significant pharmacological effects in the treatment of cardiovascular diseases. As a natural drug, Rhodiola rosea has been applied in clinical practice with little toxicity and side effects. In this paper, many experts and scholars at home and abroad have done a great deal of in-depth research and discussion on the improvement and treatment of cardiovascular diseases by Rhodiola rosea in recent ten years and found that (1) the effective medicinal ingredients of Rhodiola rosea are mainly phenyl alkyl glycosides (salidroside, tyrosol) and polysaccharides. The effective elements of Rhodiola rosea have not been fully explored, and there are still many healing mechanisms that are not clear, which still need to be studied. (2) Rhodiola has the function of multitarget and multipathway in the protection of MIRI; salidroside inhibits the expression of vascular endothelial contractile factor and promotes the expression of vascular endothelial relaxation factor by inhibiting the gene expression of HIF-1*α*, ET-1, and NOS. Rhodiola rosea inhibited the synthesis of AngII and ALD and promoted left ventricular remodeling. Inhibited the synthesis of AngII and ALD and promoted the left ventricular remodeling. In heart failure, salidroside can inhibit protein phosphorylation in PI3K/AKT/GSK3*β* pathway and protein expression of collagen-I (Col-I) and profilin-I (profilin-I) in cardiac tissue of heart failure rats, thus reducing the level of cardiac fibrosis and heart failure. (3) There are internal relations among different pathways acted by Rhodiola rosea. For example, Rhodiola can reduce the level of AngII, thereby reducing the level of myocardial fibrosis and heart failure. At the same time, fibrosis and myocardial remodeling can also cause changes in ion channels. (4) The improvement mechanism of Rhodiola on cardiovascular diseases mainly focuses on the PI3K/Akt pathway. In the protection of MIRI, salidroside can activate PI3K/Akt pathway, upregulate the protein expression of P-Akt and P-GSK-3 *β*, and protect myocardial cells from achieving the protective effect on myocardial ischemia-reperfusion injury. In antiheart failure, salidroside can inhibit the protein phosphorylation level of PI3K/AKT/GSK3*β* pathway and inhibit the protein expression of Col-I and profilin-I in heart failure rats after acute myocardial infarction (AMI), thus reducing the level of myocardial fibrosis and heart failure. (5) In the antiarrhythmia effect, the beneficial electrophysiological effects of Rhodiola rosea may be related to voltage-gated potassium channel proteins (Kv1.4, Kv1.5, Kv4.3, KvLQT1) and calcium ion channel proteins (Cav1.2); myocardial sarcoplasmic reticulum Ca^2+^ ATPase 2A (SERCA2a) is associated with activation of the PI3K/AKT channel.

In summary as shown in Table 2, Rhodiola rosea and its active ingredients can protect against myocardial ischemia, hypoxia, hypolipidemic, antithrombotic, hemodynamics, vascular function. It can be used to treat common diseases of the cardiovascular system such as coronary heart disease, hypertension, angina pectoris, myocardial infarction, heart failure, and arrhythmia. Although the components of Rhodiola rosea described in articles are not the same, the chemical constituents and effective ingredients contained in the rhizomes of different varieties of Rhodiola rosea are the same. Clinically, Rhodiola rosea has been used in fewer treatments for diseases, and there are still many dark areas in the research of Rhodiola rosea.

## Figures and Tables

**Table 1:** Components of Rhodiola rosea.

Category	Effective composition	Reference
Ketones	Quercetin, kaempferol, anthocyanin, isoquercitrin, rutin, flavonoid glycoside, cinnamic alcohol glycoside	[7]
Phenyl alkyl glycosides	Tyrosol, salidroside, rosavin, phenol glycosides...	[8] [9–11]
Coumarins	Coumarin, 7-hydroxycoumarin	[10, 11]
Organic acids	Gallic acid, myristic acid, ursolic acid, butyric acid	[12]
Polysaccharides	Arabinose, glucose, mannose, galactose, rhamnose	[10, 11]
Amino acids	Threonine, valine, leucine, isoleucine, lysine, tryptophan, phenylalanine, glycine, histidine, methionine, tyrosine, cysteine, aspartic acid, valine, proline, serine, glutamic acid, arginine	[13]
Vitamins	Vitamin A, vitamin C, vitamin D, vitamin E, vitamin B1	[11]
Inorganic elements	K, Na, Mg, Ba, Al, Ca, Cu, Fe, Zn, Sn, Mo, Mn, Cr, P, Ni, V	[12, 13]
Others	Starch, protein, fat, tannin	[9]

**Table 2:** Pharmacological effects of Rhodiola rosea.

Drugs	Dose	Animal	Model/disease	Result	Reference
Sal	300 *μ*M	Endothelial cell strain	Hypoxia model	HIF-1*α*, EF1mRNA ↓, eNOSmRNA ↑	[14]

Sal	20/40/kg/d	Wistar rat	Acute myocardial infarction model	HIF-1*α*, HIF-1*β*, and VEGF ↑	[15]

Sal	100/300 *μ*M	Endothelial cells	Endothelial dysfunction	NOX2, ROS ↓, p-eNOS ↑	[16]

Sal	20/40 mg/kg/d	SD rat	Myocardial ischemia	CK-MB, LDH, TNF-*α*, IL-6, Nox2, Nox 4, NF-*κ*BP65, P-NF-*κ*BP65, AP1 ↓	[18]

Sal	9.5 mg/kg/2 d	Rabbit	HF	IL-6, TNF-*α*, IL-1*β*, IL-8, CCL20, NLRP3, IL-17 ↓, Kir2.1, Nav1.5, NCX, PLB ↑	[42]

Sal	80 mg/kg/d	Male C57 mice	DOX-induced cardiotoxicity	Bax, Bax/Bcl-2, caspase-3 ↓, Bcl-2 ↑	[51]

Sal	600 mg/d	Breast cancer patients	Early left ventricular regional systolic dysfunction	ROS ↓, SR peak ↑	[52]

Sal	0/50/100 mg/kg/d	C57BL/6J mice	Diabetes	PGC-1*α* ↓, TFAM ↓, SIRT3 ↑	[37]

Sal	6/12/24 mg/kg/d	SD male rat	Heart failure model	LVEDD, LVESD, CL, LVMI, CVF, PVCA, hydroxyproline, RAAS ↓, LVEF, LVFS ↑	[40]

Sal	40 mg/kg/d	Male rats	MIRI model	TNF-*α*, IL-1*β* and IL-6, caspase-3, caspase-9 ↓, Bcl-2/Bax ↑	[24]

Sal	0.3/0.5 mM	VSMCs	VSMC proliferation	Drp1 ↓, Mnf2 ↑	[36]

Sal	20 mg/kg/d	Human/mice	Platelet	Platelet aggregation rate ↓, ATP ↓, C-Src, Syk, plcr2 phosphorylation ↓	[48]

Sal	12 mg/kg/d	apoE(-/-) male mice	Atherosclerotic plaque	AMP/ATP ↓	[26]

Sal	50/100/200 mg/kg/d	Wistar male rat	Goto-Kakizaki (GK) rat model of diabetes	Blood glucose, blood pressure, CaL channel ↓	[55]

Sal	40 mg/kg/d	Wistar rat	Goto-Kakizaki (GK) rat model of diabetes	Ach ↑, eNOS ↓	[54]

Sal	270 mg/kg/d	Rabbit	Heart failure model	Atrial fibrosis induction rate ↓, PI3K-AKTmRNA ↑, Kv1.4,1.5,4.3, KvLQT1, Cav1.2 ↑	[49]

Sal	25/50/100 mg/kg/d	Male mice	Type 2 diabetes model	PEPCK, glucose-6-phosphatase ↓, AMPK and PI3K/Akt, GSK3*β* ↑	[59]

Sal	50 mg/kg/d	C57BL/6J mice	High fat model	Blood glucose, IL-1*α*, IL-1*β*, IL-6 ↓, p-Akt, GSK3*β* ↓	[58]

Sal	500 *μ*M	EA.hy926 cells	Lipid oxidation and AS therapy models	Rb ↓ from G0/G1 phase to S phase	[72]

Sal	50 mg/kg/d	Male BABLc mice	HHcy mouse model	P16 ↓, p21 ↓, ICAM1 ↓, (SA)-*β*-gal ↓	[73]

Dazhu Rhodiola Rosea Capsules	75 mg/kg/d	STZ-diabetic rats	Type-1 diabetes-like model	*β*-Endorphin, GLUT 4 ↑, blood glucose, PEPCK ↓	[60]

Rhodiola rosea	100 mg/kg/d	Human cardiomyocyte strain	Oxidative stress injury model	LDH, CK, AST ↓, GSH, MDA, SOD ↑	[17]

Rhodiola-ethanol extract	0.8 g/L	STZ-diabetic rats	Heart failure	PPAR-*δ* ↑	[41]

Rhodiola-water extract	0.8 g/L	Wistar-Kyoto (WKY) rats	Spontaneously hypertensive rats model	*β*-Endorphin ↓, CIX1 ↑, SBP ↓	[50]

Tyrosol	0.1/0.25/0.5 mM	Rat	H9c2 rat cardiomyocytes	Caspase-3, cleaved caspase-8, ROS, EPK, JNK ↓, Bcl-2/Bax, Hsp70 ↑	[30]

## Data Availability

The data used to support this study are included within the article.

## Abbreviations

CVD: Cardiovascular disease
DAYS: Disability adjusted life years
Sal: Salidroside
MIRI: Myocardial ischemia-reperfusion injury
ROS: Reactive oxygen species
VEGF: Vascular endothelial growth factor
HIF-1*α*: Hypoxia-inducible factor 1*α*
HIF-1*β*: Hypoxia-inducible factor 1beta
ET-1: Endothelin-1
eNOS: Endothelial cell NO synthase
AMI: Acute myocardial infarction
OFR: Oxygen-free radicals
MDA: Malondialdehyde
LDH: Lactate dehydrogenase
GSH: Glutathione
SOD: Superoxide
C-H-R: Cold (5 degrees C)-hypoxia (428 mmHg)-restraint
TNF-*α*: Tumor necrosis factor-*α*
IL-*β*: Interleukin-1*β*
IL-6: Interleukin-6
Cyto-C: Cytochrome C
Bcl-2: B-cell lymphoma-2
Bax: Bcl-2-associated X
GSK-3*β*: Glycogen synthase kinase
Hsp70: Heat shock protein 70
EPK: Extracellular signal-regulated kinase
JNK: c-Jun amino-terminal kinase
HF: Heart failure
RAAS: Renin-angiotensin-aldosterone system
PPAR: Peroxisome proliferator-activated receptor
TC: Total cholesterol
TG: Triglycerides
LDL-C: Low-density lipoprotein cholesterol
HDL-C: High-density lipoprotein cholesterol
MDA: Malondialdehyde
BW: Bodyweight
BP: Blood pressure
FBG: Blood sugar
MPV: Mean platelet volume
PLT: Platelet count
GP: Glycoproteins
Sky: Skin tyrosine kinase
PLC*γ*2: Phospholipase C*γ*2
ALD: Aldosterone
AMPK: Adenylate-activated kinase
PEPCK: Phosphoenolpyruvate carboxy kinase
G6PD: Glucose-6-phosphatase
VSMC: Vascular smooth muscle cells
Drp1: Dynamin-related protein 1
Mfn2: Mitofusin 2
TFAM: Mitochondrial transcription factor A
SASP: Senescence-associated secretory phenotype
Rb: Retinoblastoma protein
HHcy: Hyperhomocysteine
ICAM1: Intercellular adhesion molecule 1
TNF-*α*: Tumor necrosis factor-*α*
IL: Interleukin
ISO: Isoproterenol
PGC-1*α*: Peroxisome proliferator-activated receptor-*γ* coactivator 1-*α*.

## References

[1] Dehghan M., Mente A., Zhang X. (2017). Associations of Fats and Carbohydrate Intake with Cardiovascular Disease and Mortality in 18 Countries from Five Continents (PURE): A Prospective Cohort Study. *The Lancet*.

[2] Wang C., Wang C., Liu M., Chen Z., Liu S. (2020). Temporal and spatial trends of ischemic heart disease burden in Chinese and subgroup populations from 1990 to 2016: socio-economical data from the 2016 Global Burden of Disease Study. *BMC Cardiovascular Disorders*.

[3] Francula-Zaninovic S., Nola I. (2018). Management of measurable variable cardiovascular disease’ risk factors. *Current Cardiology Reviews*.

[4] Khoury M., Manlhiot C., Gibson D. (2016). Universal screening for cardiovascular disease risk factors in adolescents to identify high-risk families: a population-based cross-sectional study. *BMC Pediatrics*.

[5] Tao H., Wu X., Cao J. (2019). Rhodiola species: a comprehensive review of traditional use, phytochemistry, pharmacology, toxicity, and clinical study. *Medicinal Research Reviews*.

[6] Panossian A., Wikman G. (2009). Evidence-based efficacy of adaptogens in fatigue, and molecular mechanisms related to their stress-protective activity. *Current Clinical Pharmacology*.

[7] Zhang S., Liu C., Bi H., Wang C. (2008). Extraction of flavonoids from Rhodiola sachlinesis A. Bor by UPE and the antioxidant activity of its extract. *Natural Product Research*.

[8] Ali Z., Fronczek F., Khan I. (2008). Phenylalkanoids and monoterpene analogues from the roots of Rhodiola rosea. *Planta Medica*.

[9] Ma G., Li W., Dou D. (2006). Rhodiolosides A-E, monoterpene glycosides from Rhodiola rosea. *Chemical & Pharmaceutical Bulletin*.

[10] Bai X., Jia X., Lu Y. (2020). Salidroside-mediated autophagic targeting of active Src and caveolin-1 suppresses low-density lipoprotein transcytosis across endothelial cells. *Oxidative Medicine and Cellular Longevity*.

[11] Tolonen A., Pakonen M., Hohtola A., Jalonen J. (2003). Phenylpropanoid glycosides from Rhodiola rosea. *Chemical & Pharmaceutical Bulletin*.

[12] Han F., Li Y., Mao X., Xu R., Yin R. (2016). Characterization of chemical constituents in Rhodiola crenulate by high-performance liquid chromatography coupled with Fourier-transform ion cyclotron resonance mass spectrometer (HPLC-FT-ICR MS). *Journal of Mass Spectrometry: JMS*.

[13] Tayade A., Dhar P., Kumar J., Sharma M., Chaurasia O., Srivastava R. (2017). Trans-Himalayan Rhodiola imbricata Edgew. root: a novel source of dietary amino acids, fatty acids and minerals. *Journal of Food Science and Technology*.

[14] Gao Q., Shao M. (2017). Salidroside improve the contractoin and dilatation function of vascular endotheliocyte. *Shaanxi Medical Journal*.

[15] Gao X., Shi H., Sun T., Ao H. (2009). Effects of Radix et Rhizoma Rhodiolae Kirilowii on expressions of von Willebrand factor, hypoxia-inducible factor 1 and vascular endothelial growth factor in myocardium of rats with acute myocardial infarction. *Journal of Chinese Integrative Medicine*.

[16] Leung S., Zhang H., Lau C., Huang Y., Lin Z. (2013). Salidroside improves homocysteine-induced endothelial dysfunction by reducing oxidative stress. *Evidence-Based Complementary and Alternative Medicine : eCAM*.

[17] Gupta V., Lahiri S., Sultana S., Tulsawani R., Kumar R. (2010). Anti-oxidative effect of Rhodiola imbricata root extract in rats during cold, hypoxia and restraint (C-H-R) exposure and post-stress recovery. *Food and Chemical Toxicology*.

[18] Zhu L., Wei T., Chang X. (2015). Effects of salidroside on myocardial injury in vivo in vitro via regulation of Nox/NF-*κ*B/AP1 pathway. *Inflammation*.

[19] Haunstetter A., Izumo S. (1998). Apoptosis. *Circulation Research*.

[20] Foo R., Mani K., Kitsis R. (2005). Death begets failure in the heart. *The Journal of Clinical Investigation*.

[21] Zhong H., Xin H., Wu L., Zhu Y. (2010). Salidroside attenuates apoptosis in ischemic cardiomyocytes: a mechanism through a mitochondria-dependent pathway. *Journal of Pharmacological Sciences*.

[22] Kvansakul M., Hinds M. (2015). The Bcl-2 family: structures, interactions and targets for drug discovery. *Apoptosis : An International Journal on Programmed Cell Death*.

[23] Raemy E., Martinou J. (2014). Involvement of cardiolipin in tBID-induced activation of BAX during apoptosis. *Chemistry and Physics of Lipids*.

[24] Zhu L., Wei T., Gao J. (2015). The cardioprotective effect of salidroside against myocardial ischemia reperfusion injury in rats by inhibiting apoptosis and inflammation. *Apoptosis : An International Journal on Programmed Cell Death*.

[25] Zuo W., Yan F., Zhang B., Hu X., Mei D. (2018). Salidroside improves brain ischemic injury by activating PI3K/Akt pathway and reduces complications induced by delayed tPA treatment. *European Journal of Pharmacology*.

[26] Xing S., Yang X., Zheng T. (2015). Salidroside improves endothelial function and alleviates atherosclerosis by activating a mitochondria-related AMPK/PI3K/Akt/eNOS pathway. *Vascular Pharmacology*.

[27] Rong L., Li Z., Leng X. (2020). Salidroside induces apoptosis and protective autophagy in human gastric cancer AGS cells through the PI3K/Akt/mTOR pathway. *Biomedicine & Pharmacotherapy*.

[28] Zhang B., Wang Y., Li H. (2016). Neuroprotective effects of salidroside through PI3K/Akt pathway activation in Alzheimer’s disease models. *Drug Design, Development and Therapy*.

[29] Zhang W., Huai Y., Miao Z. (2019). Systems pharmacology approach to investigate the molecular mechanisms of herb Rhodiola rosea L. radix. *Drug Development and Industrial Pharmacy*.

[30] Sun L., Fan H., Yang L., Shi L., Liu Y. (2015). Tyrosol prevents ischemia/reperfusion-induced cardiac injury in H9c2 cells: involvement of ROS, Hsp70, JNK and ERK, and apoptosis. *Molecules*.

[31] Tahrir F., Langford D., Amini S., Mohseni Ahooyi T., Khalili K. (2019). Mitochondrial quality control in cardiac cells: mechanisms and role in cardiac cell injury and disease. *Journal of Cellular Physiology*.

[32] Doenst T., Nguyen T., Abel E. (2013). Cardiac metabolism in heart failure. *Circulation Research*.

[33] Niemann B., Schwarzer M., Rohrbach S. (2018). Heart and mitochondria: pathophysiology and implications for cardiac surgeons. *The Thoracic and Cardiovascular Surgeon*.

[34] Kang P., Chen C., Lin P., Chilian W., Chen Y. (2017). Impairment of pH gradient and membrane potential mediates redox dysfunction in the mitochondria of the post-ischemic heart. *Basic Research in Cardiology*.

[35] Zhang W., Chen C., Wang J., Liu L., He Y., Chen Q. (2018). Mitophagy in cardiomyocytes and in platelets: a major mechanism of cardioprotection against ischemia/reperfusion injury. *Physiology*.

[36] Zhuang X., Maimaitijiang A., Li Y., Shi H., Jiang X. (2017). Salidroside inhibits high-glucose induced proliferation of vascular smooth muscle cells via inhibiting mitochondrial fission and oxidative stress. *Experimental and Therapeutic Medicine*.

[37] Li Y., Wei X., Liu S., Zhao Y., Jin S., Yang X. (2021). Salidroside protects cardiac function in mice with diabetic cardiomyopathy via activation of mitochondrial biogenesis and SIRT3. *Phytotherapy Research : PTR*.

[38] Chen P., Liu J., Ruan H. (2019). Protective effects of salidroside on cardiac function in mice with myocardial infarction. *Scientific Reports*.

[39] Ni J., Li Y., Xu Y., Guo R. (2021). Salidroside protects against cardiomyocyte apoptosis and ventricular remodeling by AKT/HO-1 signaling pathways in a diabetic cardiomyopathy mouse model. *Phytomedicine : International Journal Of Phytotherapy and Phytopharmacology*.

[40] Wu J., Yin T., Zou Q., Si L. (2016). Effects of salidroside on ventricular remodeling and renin angiotensin in chronic heart failure rats effects of aldosterone system. *Chinese Journal of Gerontology*.

[41] Cheng Y., Chen L., Lee W., Chen M., Jung Lin H., Cheng J. (2012). Increase of myocardial performance by Rhodiola-ethanol extract in diabetic rats. *Journal of Ethnopharmacology*.

[42] Hsiao Y., Tsai Y., Huang Y. (2021). Rhodiola crenulata reduces ventricular arrhythmia through mitigating the activation of IL-17 and inhibiting the MAPK signaling pathway. *Cardiovascular Drugs and Therapy*.

[43] Yang L., Li Z., Song Y. (2019). Study on urine metabolic profiling and pathogenesis of hyperlipidemia. *Clinica Chimica Acta*.

[44] Shin H., Han J., Kim H. (2014). Anti-atherosclerosis and hyperlipidemia effects of herbal mixture, Artemisia iwayomogi Kitamura and Curcuma longa Linne, in apolipoprotein E-deficient mice. *Journal of Ethnopharmacology*.

[45] Wang Z., Ma G., Wang J., Liu N. (2001). Effects of Rhodiola rosea extract on liver of rats with metabolic syndrome expression of PPAR -*α*mRNA and PPAR -*γ*mRNA. *Journal of Hebei TCM and Pharmacology*.

[46] Zhang B., Li W., Guo R., Xu Y. (2012). Salidroside decreases atherosclerotic plaque formation in low-density lipoprotein receptor-deficient mice. *Evidence-Based Complementary and Alternative Medicine : eCAM*.

[47] Liu W., Jiang C. (2015). Rhodiola crenulate capsule on unstable thrombus formation in patients with angina pectoris effect. *Drugs and Clinical*.

[48] Wei G., Xu X., Tong H. (2020). Salidroside inhibits platelet function and thrombus formation through AKT/GSK3*β* signaling pathway. *Aging*.

[49] Liu S., Hsiao Y., Chong E. (2016). Rhodiola inhibits atrial arrhythmogenesis in a heart failure model. *Journal of Cardiovascular Electrophysiology*.

[50] Lee W., Chung H., Cheng Y., Lin H., Cheng J. (2013). Rhodiola-water extract induces *β*-endorphin secretion to lower blood pressure in spontaneously hypertensive rats. *Phytotherapy Research : PTR*.

[51] Wang X., Wang X., Xiong L. (2013). Salidroside improves doxorubicin-induced cardiac dysfunction by suppression of excessive oxidative stress and cardiomyocyte apoptosis. *Journal of Cardiovascular Pharmacology*.

[52] Zhang H., Shen W., Gao C., Deng L., Shen D. (2012). Protective effects of salidroside on epirubicin-induced early left ventricular regional systolic dysfunction in patients with breast cancer. *Drugs in R&D*.

[53] Yang R., Liu K., Chen X. P. (2013). Effect of Rhodiola sacra on left ventricular remodeling and its mechanism in spontaneously hypertensive rats. *Sichuan Da Xue Xue Bao. Yi Xue Ban*.

[54] Alameddine A., Fajloun Z., Bourreau J. (2015). The cardiovascular effects of salidroside in the Goto-Kakizaki diabetic rat model. *Journal of Physiology and Pharmacology*.

[55] Ma Y., Wang J., Bai Y., Liu M., Xie M., Dai Z. (2017). Salidroside contributes to reducing blood pressure and alleviating cerebrovascular contractile activity in diabetic Goto-Kakizaki rats by inhibition of L-type calcium channel in smooth muscle cells. *BMC Pharmacology and Toxicology*.

[56] Wang X., Bao W., Liu J. (2013). Inflammatory markers and risk of type 2 diabetes. *Diabetes Care*.

[57] Donath M. (2016). Multiple benefits of targeting inflammation in the treatment of type 2 diabetes. *Diabetologia*.

[58] Wang M., Luo L., Yao L. (2016). Salidroside improves glucose homeostasis in obese mice by repressing inflammation in white adipose tissues and improving leptin sensitivity in hypothalamus. *Scientific Reports*.

[59] Zheng T., Yang X., Wu D. (2015). Salidroside ameliorates insulin resistance through activation of a mitochondria-associated AMPK/PI3K/Akt/GSK3*β* pathway. *British Journal of Pharmacology*.

[60] Niu C., Chen L., Niu H. (2014). Antihyperglycemic action of Rhodiola-aqeous extract in type1-like diabetic rats. *BMC Complementary and Alternative Medicine*.

[61] Wang S., Lu J. (2018). Protective effect of Rhodiola polysaccharide on pancreas in alloxan-induced hyperglycemic rats. *Chinese Archives of Traditional Chinese Medicine*.

[62] Song J., Wu Y., Jiang G. (2019). Sulfated polysaccharides from Rhodiola sachalinensis reduce D-gal-induced oxidative stress in NIH 3T3 cells. *International Journal of Biological Macromolecules*.

[63] Xu Y., Jiang H., Sun C. (2018). Antioxidant and hepatoprotective effects of purified Rhodiola rosea polysaccharides. *International Journal of Biological Macromolecules*.

[64] Shakeri H., Lemmens K., Gevaert A., De Meyer G., Segers V. (2018). Cellular senescence links aging and diabetes in cardiovascular disease. *American Journal of Physiology-Heart and Circulatory Physiology*.

[65] Chen Y., Yuan S., Cao Y. (2021). Gasotransmitters: potential therapeutic molecules of fibrotic diseases. *Oxidative Medicine and Cellular Longevity*.

[66] Wang Q., Shao X., Leung E., Chen Y., Yao X. (2021). Selectively targeting individual bromodomain: drug discovery and molecular mechanisms. *Pharmacological Research*.

[67] Chen Y., Shao X., Zhao X. (2021). Targeting protein arginine methyltransferase 5 in cancers: roles, inhibitors and mechanisms. *Biomedicine & Pharmacotherapy*.

[68] Wang Q., Zhang Q., Leung E., Chen Y., Yao X. (2021). Exploring the thermodynamic, kinetic and inhibitory mechanisms of 5-iTU targeting mitotic kinase haspin by integrated molecular dynamics. *Physical Chemistry Chemical Physics : PCCP*.

[69] Jafari M., Felgner J., Bussel I. (2007). Rhodiola: a promising anti-aging Chinese herb. *Rejuvenation Research*.

[70] Chen C., Song J., Chen M. (2016). Rhodiola rosea extends lifespan and improves stress tolerance in silkworm, Bombyx mori. *Biogerontology*.

[71] Bayliak M., Lushchak V. (2011). The golden root, Rhodiola rosea, prolongs lifespan but decreases oxidative stress resistance in yeast Saccharomyces cerevisiae. *Phytomedicine : international journal of phytotherapy and phytopharmacology*.

[72] Sun L., Dou F., Chen J. (2018). Salidroside slows the progression of EA.hy926 cell senescence by regulating the cell cycle in an atherosclerosis model. *Molecular Medicine Reports*.

[73] Xing S., Li J., Chen L. (2018). Salidroside attenuates endothelial cellular senescence via decreasing the expression of inflammatory cytokines and increasing the expression of SIRT3. *Mechanisms of Ageing and Development*.

[74] Steven S., Frenis K., Oelze M. (2019). Vascular inflammation and oxidative stress: major triggers for cardiovascular disease. *Oxidative Medicine and Cellular Longevity*.

[75] Kaptoge S., Seshasai S., Gao P. (2014). Inflammatory cytokines and risk of coronary heart disease: new prospective study and updated meta-analysis. *European Heart Journal*.

